# Prospective longitudinal trajectory of cancer survivorship among patients with recurrent rectal cancer: impact of treatment modalities and resection status

**DOI:** 10.1111/codi.70110

**Published:** 2025-05-15

**Authors:** Tarik Sammour, Oliver Peacock, Brian K. Bednarski, Arvind Dasari, Prajnan Das, Benny Johnson, Grace L. Smith, George J. Chang, John Skibber, Y. Nancy You

**Affiliations:** ^1^ Department of Colon and Rectal Surgery University of Texas MD Anderson Cancer Center Houston Texas USA; ^2^ Department of Gastrointestinal Medical Oncology University of Texas MD Anderson Cancer Center Houston Texas USA; ^3^ Department of Gastrointestinal Radiation Oncology University of Texas MD Anderson Cancer Center Houston Texas USA; ^4^ Department of Health Services Research University of Texas MD Anderson Cancer Center Houston Texas USA

**Keywords:** EORTC, exenteration, quality of life, rectal cancer, recurrent rectal cancer

## Abstract

**Aim:**

Recurrent rectal cancer (RRC) can be morbid and optimising cancer survivorship is a priority. The longitudinal trajectories of survivorship associated with RRC have not been prospectively depicted.

**Methods:**

We prospectively enrolled patients with RRC. Participants self‐reported quality of life (QOL) using validated European Organisation for Research and Treatment of Cancer Quality of Life Questionnaire C30 and CR29, and pain using the Brief Pain Inventory, at baseline and then every 6 months for up to 5 years or until death. Baseline scores and the longitudinal trajectory of scores were examined using linear mixed‐effects modelling.

**Results:**

Among 104 patients, 73 (70.2%) received multimodality salvage treatment with curative intent, while the remainder received best palliative treatments. Curative‐intent salvage including surgery was associated with a 30‐day operative morbidity rate of 49% and a 5‐year overall survival of 51%. Patients undergoing curative‐intent salvage versus palliative treatments did not differ in baseline QOL or pain, but the longitudinal trajectory after curative‐intent salvage showed sustained improvement of QOL and symptoms over time. This contrasted with the initial transient improvement but persistent decline with palliative treatments. Baseline QOL was significantly impacted by the anatomical site of RRC, with posterior location associated with worst QOL (*P* = 0.012). Long‐term QOL was impacted by anatomical site and status of residual tumour. Pain scores were worse among men.

**Conclusion:**

Trajectories of cancer survivorship for patients with RRC diverge, mainly influenced by anatomical site of the RCC, residual tumour status, and ability to complete curative‐intent salvage. These should inform treatment planning. Optimising selection and success of multimodality therapy remains the cornerstone for durable cancer survivorship.

**What does this paper add to the literature?**

The longitudinal trajectories of survivorship associated with recurrent rectal cancer have not been prospectively depicted. Trajectories of cancer survivorship for these patients diverge, mainly influenced by anatomical site, residual tumour status, and ability to complete curative‐intent salvage. These should inform treatment planning, selection and success for durable cancer survivorship.

## INTRODUCTION

Recurrent rectal cancer (RRC) is defined as recurrence, progression or regrowth of rectal cancer in patients who had completed prior curative‐intent treatments [1, 2]. While RCC can occur locally, systemically, or both, pelvic tumour can be associated with a high incidence of recalcitrant pelvic pain [3, 4] and can profoundly impact gastrointestinal, urinary and sexual function [5, 6]. As the experiences of patients with RRC can be characterised by considerable morbidity and eventual mortality, optimising cancer survivorship and quality of life (QOL) constitutes a critical goal in their management [7, 8, 9].

Several treatment pathways may exist for patients with RRC but differ in their relative magnitudes of intervention, morbidity risks and impact on survival and survivorship. A multimodality salvage regimen that includes surgical resection can offer a chance of cure in a subset of patients, with reported 5‐year overall survival of 28%–50% [7, 10, 11, 12, 13, 14]. However, multimodality salvage is associated with significant short‐term morbidity rates while the operative complication rates can be as high as 50% [15]. Palliative‐intent treatments can include palliative surgery, systemic chemotherapy or other best supportive care treatments.

Patient‐reported outcomes (PROs) including health‐related QOL and pain scores help depict the cancer survivorship experience and can supplement traditional clinical endpoints and aid in treatment decision making. To date, the PROs associated with RRC and with curative‐intent or palliative‐intent treatments have remained ill‐defined [16], and there are currently few longitudinal comparative data that help patients and clinicians anticipate the trajectory of their survivorship experience [17, 18, 19]. Published studies have been limited in their retrospective cross‐sectional design, heterogeneous mix of primary and recurrent cancers, absence of pre‐treatment baseline measurements, short follow‐up periods, and undefined approach to longitudinal missing data due to death or disability, which is common in this patient cohort [20].

The current study was undertaken to fill these gaps in knowledge. We aimed to prospectively measure cancer survivorship in terms of QOL and pain in patients with RRC using validated instruments and to compare the long‐term trajectories of cancer survivorship between patients who could undergo curative‐intent multimodality salvage treatment versus those who underwent palliative‐intent treatments.

## METHODS

### Patients

After approval by the University of Texas MD Anderson Cancer Center Institutional Review Board, a prospective protocol enrolled consecutive RCC patients (18 years and older) over an 8‐year study period. Patients met these inclusion criteria: biopsy‐proven recurrent adenocarcinoma in the pelvis, a history of prior curative‐intent surgical treatment at a minimum interval of 3 months prior to presentation, provided consent to, and had sufficient command of, English language for longitudinal survey completion. Patients with synchronous metastatic disease were eligible. Exclusion criteria included concurrent second malignancy, pre‐existing documented chronic pain syndrome, chronic constipation or dysmotility (unrelated to cancer or obstruction), prior non‐operative management for rectal carcinoma.

### Treatment and clinical data

All patients were treated in a specialised colorectal cancer multidisciplinary setting as per standard clinical care. All data regarding patient demographics, tumour staging, salvage surgery and postoperative recovery, pathology, as well as neoadjuvant and adjuvant treatments were recorded prospectively. Patients who underwent surgical exploration with complete resection of gross tumour (R0/R1 margin status) were considered to have had surgery with curative intent. Intraoperative radiotherapy has been utilised in selected patients with close R0 or R1 resection margin, typically based on surgeon's intraoperative judgement and/or intraoperative frozen pathology assessment [21]. The palliative‐intent treatment group included patients with gross tumour at the end of a surgical intervention and/or those who opted to receive or received best systemic and other supportive therapy. Postoperative complications were stratified by the Clavien–Dindo classification system, and all events potentially attributable to the operation were recorded without a limit to the time frame postoperatively [22]. Oncological outcomes, including local or distant disease recurrence, and death were prospectively recorded and subsequently re‐confirmed by retrospective chart review to ensure up‐to‐date follow‐up data.

### Patient‐reported cancer survivorship outcomes

Patients’ self‐reported QOL and pain scores were captured prospectively and longitudinally. Participants were self‐administered validated instruments during clinic visits by a dedicated study coordinator who was blinded to the patients' clinical status. Generic QOL was measured by the validated European Organisation for Research and Treatment of Cancer (EORTC) Quality of Life Questionnaire C30 (QLQ‐C30) and the colorectal‐cancer‐disease‐specific QOL by the EORTC QLQ‐CR29. Pain was assessed using the validated Brief Pain Inventory (BPI) questionnaire [23, 24]. The QOL questionnaires were administered at baseline and then at 6‐month intervals for up to 5 years, or until death. The BPI questionnaire was administered at baseline and then at 6‐month intervals for up to 3 years, or until death.

QOL was globally defined as the patient's appraisal of, and satisfaction with, their current level of functioning over the past 4 weeks, compared to what they perceive to be possible or ideal [25]. The QLQ‐C30 survey includes 30 generic QOL questions on a Likert 4‐point scale constituting a global health status scale, five functional scales, three symptom scales and six single items. These questions address daily self‐care activities, appetite, sleep patterns, emotional state and overall perceived state of health [26]. The QLQ‐CR29 survey includes 29 disease‐specific questions on a similar Likert 4‐point scale. It focuses on issues related to colorectal cancer, such as symptoms (gastrointestinal, urinary, pain and others) and functions (sexual, body image and others) [23, 27]. Three summary scores were calculated according to standardised scoring procedures: a function scale, a symptom scale and a global health status scale. Scores are standardised to a scale of 0–100 through linear transformation. A higher score in global or functioning scales represents a higher (‘better’) level of QOL, while a higher score in the symptom scales represents a higher (‘worse’) level of symptoms.

The BPI survey includes both text and pictorial representations of a patient's pain level as well as the degree of interference with daily activities caused by pain [28]. The short and long forms of the BPI were used as per recommended guidelines [24].

### Statistical analysis

Continuous variables were examined for parametricity using the Kolmogorov–Smirnov test. Parametric continuous data were presented as mean (standard deviation) and non‐parametric continuous data as median (interquartile range). Group comparisons were made using the *χ* ^2^ (or Fisher's exact) test for categorical variables and the *t* test or the Mann–Whitney *U* test for parametric or non‐parametric continuous variables. Median follow‐up was estimated using the Kaplan–Meier method.

While this study is descriptive, it was estimated that inclusion of 100 evaluable patients would enable meaningful comparison of patients who underwent curative versus palliative surgery, detecting an effect size of at least 0.78 using a two‐sided *t* test, with a type I error of 0.05 and type II error of 20% (effect size calculated as the absolute difference between groups in terms of change of QOL divided by the common standard deviation) [4]. To be evaluable, a patient must have completed a minimum of two questionnaires, one at baseline and another at any time after baseline. All sample size calculations were carried out using nQuery Advisor 7.0.

The longitudinal trajectory of QOL and BPI scores over time was assessed using linear mixed‐effects modelling [29]. We assumed a random intercept and slope for each patient, and used the unstructured variance–covariance matrix to consider the correlation within each subject and account for missing data points. Statistical significance was accepted at the 0.05 level. Data were analysed using IBM SPSS Statistics for Windows, Version 22.0 (IBM Corp, Armonk, NY, USA) and SAS 9.3 (SAS Institute Inc., Cary, NC, USA).

## RESULTS

**FIGURE 1:**
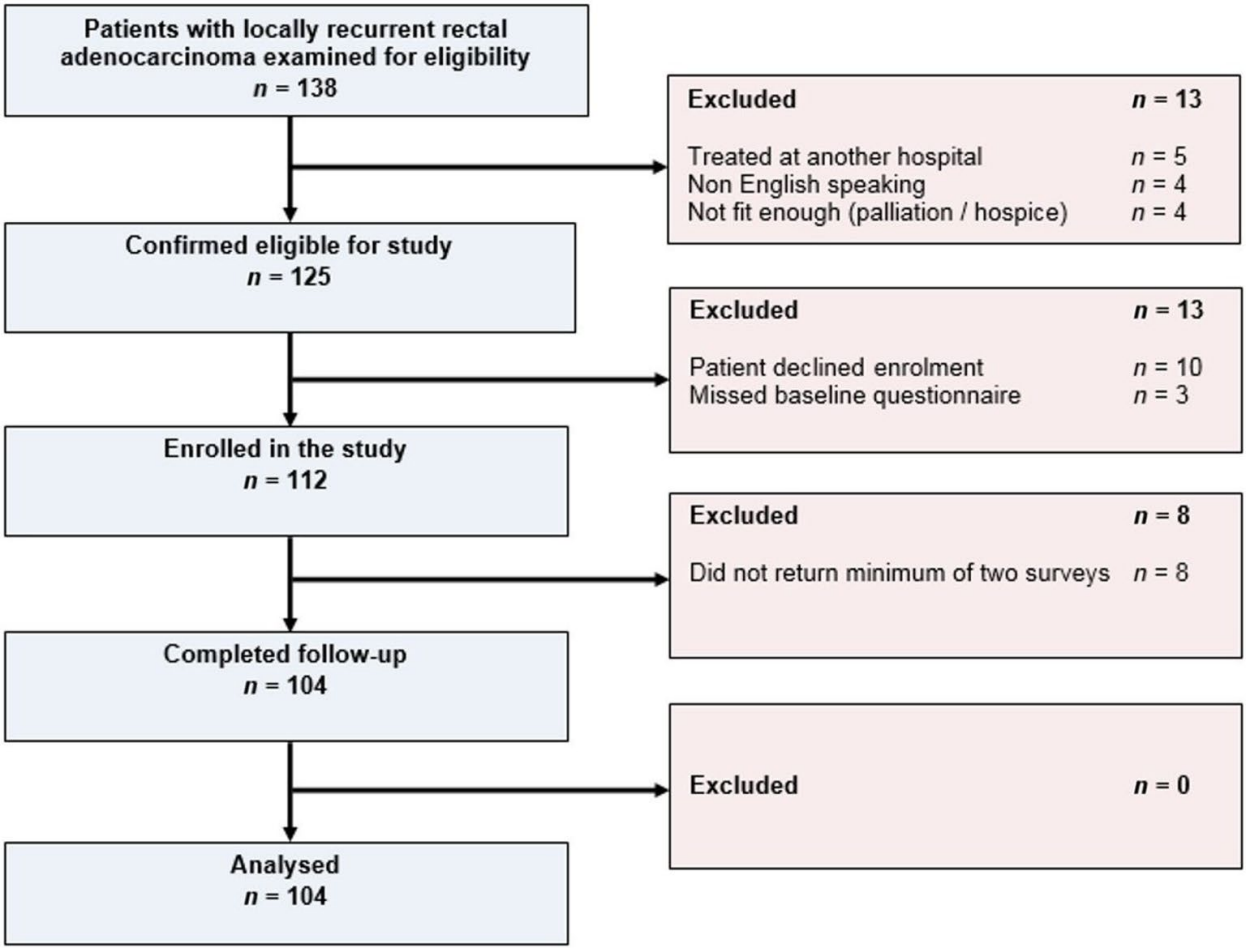
Patient flow diagram.

Among 104 evaluable patients with RRC (Figure 1), 73 (70.2%) underwent curative‐intent multimodality salvage treatment and 31 (29.8%) received palliative treatments (Table 1). The median follow‐up was 41 months (42 months in the curative, and 36 months in the palliative group).

**TABLE 1:** Characteristics of 104 patients with recurrent rectal cancer by treatment intent.

	All patients	Curative intent	Palliative intent	*P*
	(*n* = 104)	(*n* = 73)	(*n* = 31)	
Median age in years (IQR)	56 (18)	56 (20)	57 (11)	0.376
Sex				
Female	44 (42)	33 (45)	11 (36)	0.393
Male	60 (58)	40 (55)	20 (65)	
Ethnicity				
Caucasian	77 (74)	56 (77)	21 (68)	0.242
Black	8 (8)	7 (10)	1 (3)	
Asian	2 (2)	1 (1)	1 (3)	
Hispanic	17 (16)	9 (12)	8 (26)	
Recurrence anatomy				
Central compartment/anastomotic	19 (18)	12 (16)	7 (23)	**0.010**
Anterior compartment	26 (25)	25 (34)	1 (3)	
Posterior compartment	22 (21)	14 (19)	8 (26)	
Lateral compartment/other	37 (36)	22 (30)	15 (48)	
Survival (5 year estimated)				
Overall	38.7%	50.9%	7.7%	**<0.0001**.
Cancer specific	46.2%	59.6%	11.5%	**<0.0001**

*Note*: Data presented as *n* (%) unless otherwise stated. *P* value < 0.05 considered significant.

Abbreviation: IQR, interquartile range.

### Patient characteristics, treatment details, perioperative and oncological outcomes

Patients who underwent curative‐intent salvage versus palliative treatments did not differ in age, sex or race/ethnicity (Table 1). Among patients who underwent curative‐intent salvage, they were more likely to have disease located in the anterior pelvic compartment (Table 1). R0 resection margin was achieved in 75% of the patients after multimodality therapy and repeat pelvic operations where a majority (67%) involved multi‐visceral resection. Surgical salvage was associated with a complication rate of 49% (14% Grade 3 and 7% Grade 4). Postoperatively, 12% of the patients were admitted to the intensive care unit. Approximately one in five (21%) patients required home health assistance or transitional care after discharge from the hospital (Table 2).

**TABLE 2:** Treatment details and outcomes of recurrent rectal cancer patients treated with curative‐intent multimodality salvage regimen.

	Curative intent (*n* = 73)
Type of surgery	
Ultralow anterior resection/abdominoperineal resection	24 (33)
Posterior pelvic exenteration	12 (16)
Total pelvic exenteration	19 (26)
Concomitant bone resection including sacrectomy	19 (26)
Concomitant lateral compartment resection/other	2 (3)
Intraoperative radiotherapy	35 (48)
Postoperative adjuvant chemotherapy	60 (82)
Resection margin	
No residual tumour (R0/R1)	73 (100)
Tumour at margin (R2)	0 (0)
Complication by grade	
None	37 (51)
I/II	21 (48)
III	10 (14)
IV	5 (7)
ICU admission	9 (12)
Home health/transitional care after discharge	15 (21)
Readmission within 30 days	14 (19)
Median hospital day stay (IQR)	8 (5)
Disease recurrence at 5 years	44 (60)
Local only	14 (19)
Distant only	19 (26)
Both local and distant	11 (15)
Median time to second recurrence in months (IQR)	14 (21)

*Note*: Data presented as *n* (%) unless otherwise stated.

Abbreviations: ICU, intensive care unit; IQR, interquartile range.

The estimated 5‐year overall and cancer‐specific survival rates were significantly higher in the curative‐intent group (Table 1). Forty‐four patients developed a recurrence at a median of 14 months (Table 2). Among them, the majority (68.2%) developed distant metastases with or without concomitant local re‐recurrence (Table 2).

### Patient‐reported outcomes at baseline

Baseline pain severity and interference scores did not differ between patients who underwent curative‐intent salvage versus palliative treatments (Table 3). Male patients experienced more severe pain at baseline. Baseline pain scores did not significantly correlate with perioperative outcomes.

**TABLE 3:** Prospectively collected pain and quality of life scores at baseline and associated factors.

Characteristic	Brief pain inventory				EORTC QLQ‐C30						EORTC QLQ‐CR29			
	Pain severity	*P*	Pain interference	*P*	Function scale	*P*	Symptom scale	*P*	Global health	*P*	Function scale	*P*	Symptom scale	*P*
Sex														
Female	2.7 (1.9)	0.020	3.1 (2.4)	0.162	74.6 (19.4)	0.662	24.7 (15.7)	0.824	65.7 (20.9)	0.658	63.5 (18.1)	0.346	19.8 (14.4)	0.636
Male	**3.9 (2.1)**		4.0 (2.7)		76.3 (19.8)		23.9 (18.1)		67.6 (23.2)		59.9 (19.0)		18.6 (12.0)	
Ethnicity														
Caucasian	3.4 (2.2)	0.346	3.5 (2.7)	0.200	73.7 (20.0)	0.363	24.9 (17.2)	0.917	66.2 (22.6)	0.940	63.0 (18.3)	0.128	19.4 (13.7)	0.959
African American	3.1 (2.0)		2.4 (1.6)		82.4 (22.6)		23.6 (19.4)		65.6 (19.6)		63.9 (4.1)		19.0 (10.5)	
Asian	2.6 (0.2)		2.6 (1.4)		86.8 (16.3)		23.1 (17.0)		70.8 (5.9)		70.8 (4.2)		16.0 (3.3)	
Hispanic	4.2 (1.9)		4.8 (2.3)		80.0 (15.6)		21.7 (16.6)		69.6 (23.9)		51.6 (21.5)		17.9 (12.0)	
Recurrence anatomy														
Central/anastomotic	2.7 (1.8)	0.387	3.8 (3.2)	0.731	72.5 (24.4)	0.012	25.9 (18.7)	0.065	**61.0 (25.5)**	0.014	61.3 (17.3)	0.212	15.8 (10.8)	0.070
Anterior	3.1 (1.9)		3.0 (2.4)		85.4 (11.1)		17.6 (14.1)		79.0 (16.2)		68.2 (21.9)		15.8 (9.0)	
Posterior	3.9 (2.1)		4.0 (2.6)		**67.4 (22.9)**		30.7 (20.2)		62.1 (23.4)		57.3 (16.5)		24.7 (16.8)	
Lateral/other	3.7 (2.3)		3.7 (2.6)		75.5 (16.8)		24.1 (14.9)		64.4 (20.9)		59.5 (17.7)		19.7 (13.0)	
Curative‐intent surgery														
Yes	3.3 (2.3)	0.349	3.3 (2.7)	0.092	75.9 (19.8)	0.788	23.9 (17.9)	0.757	68.3 (20.3)	0.363	62.8 (19.0)	0.229	18.5 (13.3)	0.504
No	3.8 (1.7)		4.3 (2.2)		74.8 (19.3)		25.0 (15.1)		63.4 (26.1)		58.1 (17.5)		20.4 (12.5)	
Type of surgery^a^														
ULAR/APR	3.8 (2.8)	0.527	4.0 (3.1)	0.459	70.6 (26.0)	0.372	25.3 (20.4)	0.679	66.7 (22.6)	0.196	60.9 (17.1)	0.474	18.7 (14.7)	0.662
Posterior pelvic exent	2.6 (1.9)		3.0 (1.8)		78.5 (15.4)		18.9 (13.8)		71.5 (14.4)		70.8 (16.3)		14.0 (8.6)	
Total pelvic exent	2.8 (2.3)		2.0 (2.4)		82.9 (14.1)		22.3 (17.5)		75.0 (18.0)		57.5 (27.2)		19.0 (10.1)	
Bone resection	6 (0)		4.3 (0)		66.3 (24.5)		16.4 (14.4)		41.7 (11.8)		61.1 (7.9)		14.4 (17.4)	
Lateral/other	3.4 (1.6)		3.8 (3.0)		76.1 (16.8)		27.7 (18.3)		65.4 (21.4)		64.4 (15.8)		21.3 (16.0)	
Resection margin^a^														
Negative (>1 mm)	3.4 (2.1)	0.512	3.5 (2.9)	0.790	75.7 (20.3)	0.288	24.8 (19.3)	0.064	72.5 (20.2)	0.050	66.3 (18.3)	0.359	18.8 (13.1)	0.834
Positive (<1 mm)	4.3 (2.3)		3.3 (1.5)		81.3 (11.4)		15.4 (10.5)		**57.3 (17.5)**		60.1 (16.7)		19.6 (9.0)	
Post‐op complication^a^														
None	3.2 (2.2)	0.988	2.9 (2.3)	0.605	79.0 (12.1)	0.898	22.9 (16.8)	0.928	73.6 (18.7)	0.211	60.4 (20.7)	0.111	16.3 (10.9)	0.566
I	3.9 (2.4)		2.4 (1.6)		74.7 (15.7)		25.7 (21.3)		63.9 (4.8)		50.0 (8.3)		16.6 (3.2)	
II	3.4 (2.0)		4.2 (3.2)		74.1 (24.0)		27.0 (21.4)		73.0 (22.5)		62.6 (17.1)		23.4 (16.0)	
III	3.5 (3.2)		2.7 (2.2)		74.3 (16.7)		20.8 (15.9)		57.5 (17.8)		75.3 (16.4)		20.6 (17.0)	
IV	3.8 (2.7)		2.0 (2.2)		79.3 (19.8)		23.8 (14.7)		63.3 (19.2)		49.4 (25.5)		19.4 (4.6)	
Home health/transition care post‐op^a^														
Yes	3.7 (2.8)	0.655	3.5 (2.6)	0.701	75.0 (23.4)	0.866	27.3 (19.5)	0.830	64.4 (19.3)	0.644	56.3 (21.2)	0.274	21.5 (12.1)	0.952
No	3.3 (1.9)		3.1 (2.4)		73.9 (17.5)		26.1 (17.9)		67.2 (22.0)		63.5 (18.8)		21.8 (15.2)	
Readmission^a^														
Yes	3.6 (3.0)	0.856	3.6 (2.5)	0.722	77.0 (15.1)	0.539	23.7 (14.8)	0.536	66.7 (18.8)	0.929	63.5 (22.7)	0.698	18.2 (7.4)	0.163
No	3.4 (2.0)		3.2 (2.5)		73.9 (20.2)		26.7 (19.3)		67.2 (22.4)		60.9 (18.3)		22.5 (15.8)	
Death (all cause)^a^														
Yes	3.5 (2.8)	0.758	2.9 (2.7)	0.587	74.9 (23.4)	0.652	24.2 (19.4)	0.870	69.2 (21.8)	0.920	62.2 (20.9)	0.810	22.2 (13.0)	0.059
No	3.2 (2.0)		3.4 (2.8)		77.4 (17.8)		23.4 (17.8)		68.7 (19.7)		63.5 (18.2)		15.8 (12.4)	
Death (cancer specific)^a^														
Yes	3.5 (2.7)	0.789	3.0 (2.3)	0.713	74.4 (21.6)	0.634	24.6 (17.5)	0.810	66.7 (23.6)	0.652	60.6 (22.5)	0.616	23.4 (14.8)	0.080
No	3.3 (2.1)		3.3 (2.9)		77.2 (19.2)		23.4 (18.5)		69.6 (19.2)		63.8 (18.0)		16.2 (11.8)	
Recurrence^a^														
Yes	3.5 (2.3)	0.459	3.4 (2.8)	0.669	76.2 (19.8)	0.900	24.5 (17.2)	0.678	**64.7 (20.0)**	0.021	61.9 (18.8)	0.576	20.2 (14.0)	0.079
No	2.9 (2.2)		3.0 (2.7)		75.5 (20.2)		22.5 (19.8)		76.5 (19.0)		64.8 (19.8)		14.8 (10.7)	

*Note*: All results are presented as mean (SD). *P* value < 0.05 considered significant.

Abbreviations: APR, abdominoperineal resection; EORTC, European Organisation for Research and Treatment of Cancer; exent, exenteration; post‐op, postoperative; QLQ, Quality of Life Questionnaire; ULAR, ultralow anterior resection.

^a^ Only patients undergoing surgery with curative intent included in this analysis.

Patients undergoing curative‐intent salvage versus palliative treatments also did not differ in their baseline generic or disease‐specific QOL, whether in global health, function or symptom scores (Table 3). Baseline generic QOL scores correlated with anatomy of the RRC: those with central or anastomotic recurrence reported these lowest global health scores, while those with posterior compartment recurrence reported significantly worse function (Table 3). In patients who underwent curative‐intent salvage, lower baseline global QOL scores correlated with having an R1 resection margin (<1 mm) and ultimately a re‐recurrence (Table 3).

### Longitudinal trajectory of patient‐reported outcomes

**FIGURE 2:**
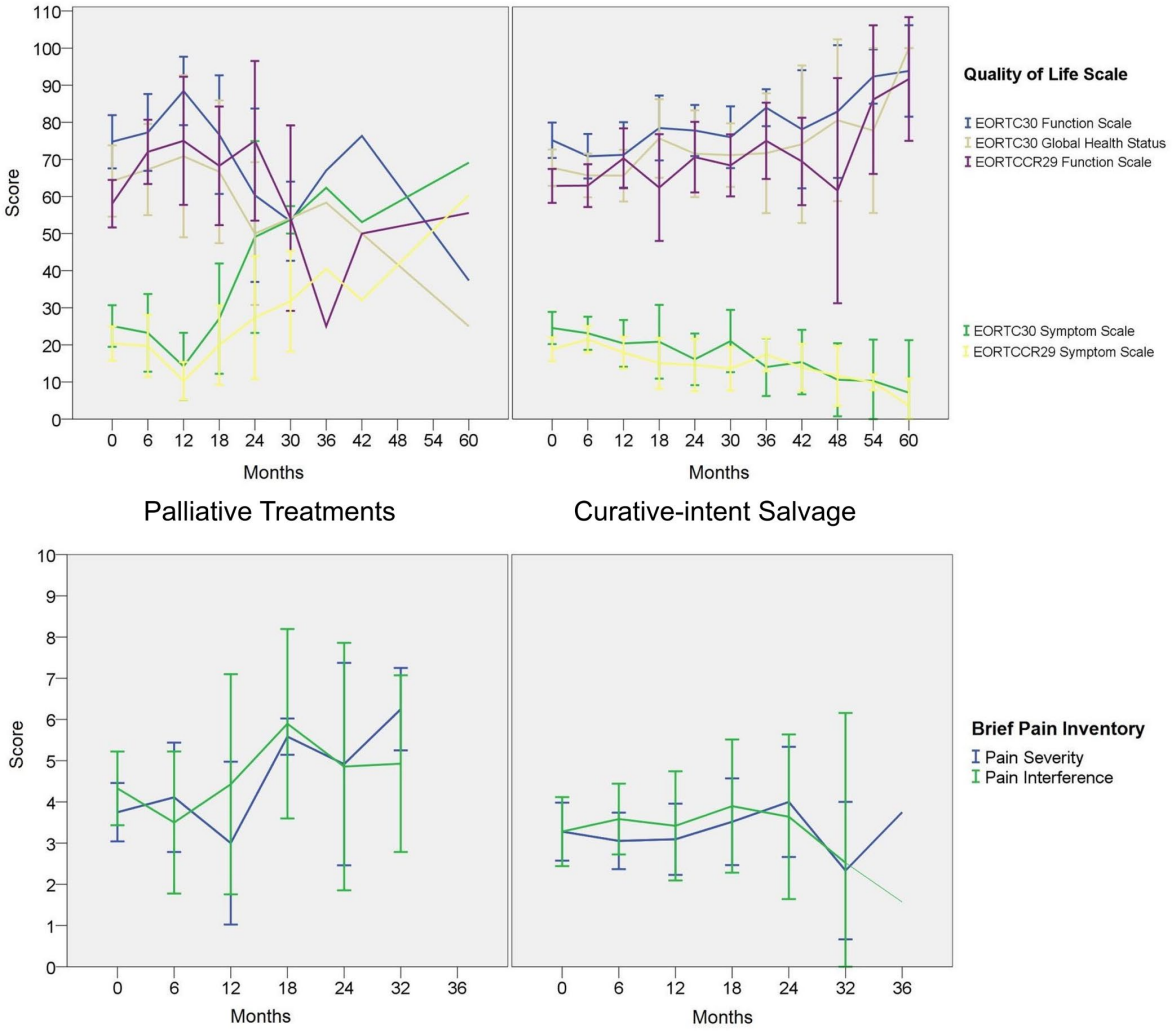
Trajectory of quality of life (EORTC‐C30, EORTC‐CR29) and pain (Brief Pain Inventory) in patients managed with curative‐intent salvage versus those who were not. Score is expressed as mean with SE in error bars. Function scales, global health, higher score is better. Symptom scales, for both pain scales, higher is worse.

The longitudinal trajectory of cancer survivorship for RRC patients as measured by QOL and pain scores is depicted in Figure 2. After curative‐intent salvage surgery, QOL scores, including both generic and disease‐specific global and function scales, demonstrated gradual sustained improvement over time. In contrast, palliative treatments resulted in early but transient improvement in QOL, with a persistent downward trend after 12 months. Pain scores did not demonstrate statistically significant fluctuation over time.

Longitudinal mixed modelling analysis of QOL scores showed that the worst generic function and symptom scores were associated with a posterior recurrence (Table 4). Furthermore, poor global health score longitudinally was associated with a positive resection margin (Table 4).

**TABLE 4:** Longitudinal analysis of pain and quality of life scores (linear mixed modelling, fixed effects).

Characteristic	Brief pain inventory				EORTC QLQ‐C30						EORTC QLQ‐CR29			
	Pain severity	*P*	Pain interference	*P*	Function scale	*P*	Symptom scale	*P*	Global health	*P*	Function scale	*P*	Symptom scale	*P*
Sex														
Female	2.7 (0.2)	0.019	3.2 (0.3)	0.086	75.0 (1.7)	0.843	24.1 (1.5)	0.617	66.5 (1.9)	0.620	65.4 (1.8)	0.268	18.5 (1.1)	0.671
Male	**3.8 (0)**		4.1 (0.2)		76.6 (1.4)		21.1 (1.3)		68.6 (1.6)		64.9 (1.5)		17.9 (0.9)	
Ethnicity														
Caucasian	3.3 (0.2)	0.187	3.7 (0.2)	0.313	75.7 (1.2)	0.254	22.4 (1.1)	0.758	67.4 (1.4)	0.431	66.4 (1.3)	0.118	18.0 (0.8)	0.725
African American	3.6 (0.5)		3.9 (0.6)		70.7 (4.5)		28.6 (3.8)		65.9 (4.8)		67.2 (4.5)		20.5 (2.9)	
Asian	1.6 (1.5)		2.6 (1.9)		89.4 (11.5)		17.3 (9.7)		72.4 (12.6)		72.1 (10.9)		15.0 (7.6)	
Hispanic	3.8 (0)		3.6 (0.5)		78.6 (3.3)		18.9 (2.9)		69.8 (3.6)		54.1 (3.4)		17.8 (2.2)	
Recurrence anatomy														
Central/anastomotic	2.9 (0.4)	0.305	3.9 (0.6)	0.444	75.1 (2.7)	0.039	23.3 (2.5)	0.042	63.1 (3.1)	0.151	59.4 (2.9)	0.393	17.5 (1.8)	0.062
Anterior	2.8 (0.3)		2.9 (0.4)		83.0 (2.1)		16.8 (1.9)		76.6 (2.4)		70.5 (2.3)		14.2 (1.4)	
Posterior	3.9 (0.3)		4.2 (0.4)		**68.5 (2.1)**		**29.2 (2.0)**		64.2 (2.5)		65.6 (2.4)		21.8 (1.5)	
Lateral/other	3.8 (0)		3.9 (0.3)		76.3 (1.8)		21.3 (1.7)		66.5 (2.0)		64.2 (1.9)		18.6 (1.2)	
Curative‐intent surgery														
Yes	3.2 (1.7)	0.047	3.5 (0.2)	0.049	76.7 (1.2)	0.744	20.9 (1.1)	0.496	69.0 (1.4)	0.466	65.7 (1.3)	0.712	17.3 (0.8)	0.812
No	**4.1 (0.3)**		**4.4 (0.4)**		73.5 (2.3)		26.9 (2.1)		63.6 (2.6)		63.2 (2.4)		20.9 (1.5)	
Type of surgery^a^														
ULAR/APR	3.7 (0.3)	0.686	4.7 (0.4)	0.803	73.4 (2.3)	0.389	24.1 (2.1)	0.719	69.4 (2.5)	0.338	59.6 (2.5)	0.497	19.5 (1.6)	0.779
3\posterior pelvic exent	2.8 (0.5)		3.0 (0.6)		79.1 (2.7)		15.1 (2.4)		70.5 (3.2)		68.1 (3.1)		14.5 (1.8)	
Total pelvic exent	2.7 (0.4)		2.7 (0.5)		83.5 (2.3)		16.2 (2.0)		76.2 (2.8)		70.4 (2.7)		15.2 (1.5)	
Bone resection	3.8 (0)		1.7 (0.3)		64.4 (7.4)		24.9 (6.3)		50.0 (7.1)		62.8 (7.9)		18.8 (4.5)	
Lateral/other	2.9 (0.3)		3.3 (0.4)		75.7 (2.1)		21.8 (1.9)		65.9 (2.3)		68.5 (2.3)		17.7 (1.4)	
Resection margin^a^														
R0/R1	3.4 (0.2)	0.433	3.2 (0.3)	0.707	78.2 (1.3)	0.323	19.8 (1.3)	0.051	70.4 (1.6)	0.024	66.7 (1.5)	0.299	16.6 (0.9)	0.374
R2	3.0 (0.3)		4.0 (0.4)		72.5 (2.7)		24.0 (2.3)		**61.9 (2.7)**		60.0 (2.7)		21.0 (1.7)	
Complication by grade^a^														
None	2.8 (0.4)	0.942	2.4 (0.4)	0.332	78.2 (2.2)	0.383	19.8 (1.9)	0.306	72.7 (2.5)	0.137	63.4 (2.3)	0.095	17.8 (1.4)	0.543
I	2.9 (0.7)		3.7 (0.8)		77.5 (7.6)		20.2 (6.0)		66.4 (7.6)		58.7 (7.2)		16.0 (4.8)	
II	3.1 (0.3)		3.7 (0.4)		74.9 (2.2)		24.1 (2.0)		67.6 (2.6)		60.8 (2.2)		19.3 (1.4)	
III	3.8 (0)		2.9 (0.4)		80.5 (2.5)		14.0 (2.1)		67.4 (3.2)		79.4 (2.6)		14.3 (1.5)	
IV	3.5 (0.7)		4.2 (0.8)		66.1 (6.5)		40.3 (5.5)		56.3 (6.6)		47.3 (6.3)		29.3 (4.3)	

*Note*: All results are presented as estimated marginal mean (SE). *P* value < 0.05 considered significant.

Abbreviations: APR, abdominoperineal resection; EORTC, European Organisation for Research and Treatment of Cancer; exent, exenteration; post‐op, postoperative; QLQ, Quality of Life Questionnaire; ULAR, ultralow anterior resection.

^a^ Only patients undergoing surgery with curative intent included in this analysis.

The longitudinal trajectory of pain scores demonstrated that, over time, palliative‐intent treatment was associated with a near‐significant trend toward increased pain severity (*P* = 0.05) and interference (*P* = 0.05) scores (Table 4). The gender difference in pain severity identified at baseline also persisted on longitudinal analysis, with men consistently reporting worse pain severity (*P* = 0.02; Table 4).

## DISCUSSION

We herein have prospectively profiled the longitudinal projection of cancer survivorship for patients with RRC facing different treatment pathways. While patients receiving curative‐intent versus palliative treatments did not differ in their baseline QOL or pain measures, their longitudinal trajectories diverged significantly. Curative‐intent multimodality salvage was associated with a gradual but sustained improvement in QOL along with diminishing symptoms, whereas in patients receiving palliative treatments both QOL and symptoms deteriorated over time after an initial period of amelioration (Figure 2). In addition, we specifically identified that the anatomical distribution of the RRC significantly influenced QOL at baseline and throughout survivorship, while positive resection margin negatively influenced long‐term QOL. Finally, a significant determinant of pain at baseline and longitudinally was male gender. Taken together, our knowledge about longitudinal cancer survivorship has highlighted the additional sustained value of curative‐intent multimodality salvage for RRC that extends beyond traditional survival outcomes.

This largest single‐centre prospective analysis of cancer survivorship in the unique population of patients with RRC aimed to fulfil a significant gap in knowledge. In our prior studies, pain and QOL were identified as two key interrelated elements of survivorship. We had correlated greater baseline pain with more postoperative pain and even with overall survival [3, 20]. In the current study, we focused on depicting the longitudinal survivorship experience. We significantly expanded our patient cohort and utilised well‐standardised and validated EORTC assessment tools [30]. Compared to other generic QOL instruments, EORTC tools are more widely validated and place a larger emphasis on disease symptoms and treatment consequences which are highly relevant to cancer survivorship [27]. Additionally, the more recently developed QLQ‐CR29 survey may be more responsive in patients with advanced colorectal cancer [26] and captures newer and more relevant QOL concepts [23, 27].

Our study corroborates prior reports that generic QOL scores improved over time after curative‐intent salvage surgery. In a multicentre study, postoperative QOL was measured at 12‐month intervals using Functional Assessment of Cancer Therapy—Colorectal, the Assessment of Quality of Life and the SF‐6D tools [31]. A subgroup analysis of 117 patients with RRC showed a sharp drop in QOL at 1 month after surgery, with a rapid improvement over at 3 months and sustained improvement at 12 months [31]. Additionally, our current study significantly strengthens the conclusion that resection margin status is a critical determinant of long‐term QOL. Indeed, other studies examining QOL among patients who had undergone pelvic exenteration for different diseases [18, 31, 32, 33, 34] had suggested that the improvement in QOL measures after exenteration was associated with curative‐intent but not palliative‐intent resections [34]. However, most were not specific to patients with RRC, leaving depiction of disease‐specific cancer survivorship a yet unfilled gap in knowledge [20]. A systematic review identified only 105 patients with RRC undergoing a mixture of curative (67%) and palliative (23%) surgery [20]. Retrospective data in this study showed a high level of baseline bleeding, obstructive and pain symptoms, an improvement after surgery, and a relapse or new development of symptoms in a majority of patients over time [35]. However, QOL was not formally measured and how these trends differed between curative‐intent versus palliative‐intent surgical patients was not studied [35]. Finally, in a prospective study of 45 RRC patients evaluated with EORTC QLQ‐C30 at baseline, 1 and 3 years after surgery, resection margin of R0/R1 status significantly predicted QOL improvement over time; indeed, at 3 years, the scores for R0 resection patients were equivalent to those of control subjects with curatively treated primary rectal cancer [19].

A key determinant of curative‐intent salvage surgery with negative margins is disease anatomy. Our analysis demonstrated that disease anatomy significantly impacted QOL both at baseline and in the long term, where patients with RRC predominantly in the posterior compartment fared worse than patients with disease in other compartments. This finding is consistent with the accepted understanding that posterior RRC with sacral invasion is challenging to treat surgically depending on the level of invasion [36, 37]. While sacrectomy can be curative, it is associated with considerable morbidity and the risk of chronic pain [38]. In addition, it is notable that while patients with central or anastomotic compartment recurrence experienced similarly low global QOL scores as those with posterior compartment disease at baseline, this difference did not persist over time. The association between worse QOL and margin‐positive resection both at baseline and longitudinally could be attributed to a bias in the proportion of patients with disease anatomy who are less amendable to wider margin‐negative curative surgery, or to a potential toward a higher rate of disease re‐recurrence associated with margin‐positive resection [7, 38]. Taken together, the anatomy of RRC and the ability to achieve margin‐negative curative‐intent salvage are related and key determinates of survivorship [34]. Further, these factors can be regarded as non‐modifiable risk factors for poor long‐term cancer survivorship, and our findings reported herein should serve to further inform treatment selection and informed consent discussions.

Our finding of worse pain scores in male patients is intriguing. Several possible reasons could be speculated. In contrast to a wider female pelvis where the presence of a uterus may afford relative protection against anterior genitourinary invasion [39], the narrow anatomy of the male pelvis could leave less room to accommodate the mass effect of RRC. Furthermore, the lack of anterior protection in the male pelvis may allow RRC to directly invade the genitourinary structures more readily [40]. While a subjective element to the gender‐specific difference in reported pain may be present, the chronic pain literature suggests that pain scores tend to actually be worse in female patients, both in cancer and non‐cancer settings [41, 42]. In a recent study of colorectal cancer survivors, worse pain severity and interference were observed in female patients both at baseline and long term [43]. While gender‐specific biological differences or treatment bias may underlie self‐reported pain [44], our findings of worse pain in men suggested that there were probably factors specific to patients with RRC, most likely related to the biological anatomical differences and anatomy for RRC.

In this delineation of long‐term cancer survivorship for a single disease, despite unique strengths of prospective data collection, long‐term follow‐up and robust statistical analyses, several limitations remain. First, a longitudinal drop‐out rate was substantial at 24%, chiefly due to disability, mortality and possibly survey fatigue with our relatively long duration of follow‐up (41 months). The study cohort was recruited and enrolled over an 8‐year study period, and there is currently no international consensus on what the time frame since index curative‐intent operation is best to define pelvic recurrence. Our choice of 3 months was intended to be inclusive. The selection criteria and the operative principles had not changed over this time. Nonetheless, patients who otherwise met our inclusion criteria based on their disease history may feel overwhelmed by their treatment and decline to participate in prospective assessments, leading to enrolment bias. In addition, since respondents with improved QOL following curative‐intent salvage and those with progressively declining QOL with palliative treatments were both included, survivor bias and responder bias were probably unable to be fully accounted for in our study. Second, the instruments used for pain and QOL, although validated and specific to colorectal cancer, were not specific for RRC. However, until such a disease‐specific tool is developed and validated, the EORTC instruments remain the most accurate and available method for measuring PROs [20]. The inclusion of other specific pelvic organ function questionnaires were strongly considered, but these EORTC instruments were chosen after balancing the principles of parsimony, minimising patient burden and facilitating response return. Indeed, an ongoing international effort aimed at developing a validated patient‐reported outcome measure specifically for patients with pelvic RCC hold promise to significantly advance the field [45]. Finally, one could question whether information on post‐treatment QOL can be used in preoperative or intraoperative patient decision making. The relation between post‐treatment PROs, and preoperative decisional conflict and tradeoff is a complex and nonlinear one [46]. Similarly, our data are insufficient to provide information regarding any alteration in QOL that may or may not be associated with or attributable to intraoperative radiation therapy. Nevertheless, it would be critical to interpret the data gained from this study in corroboration with data from previous QOL studies, in the context of individual patients' preferences, and to help inform shared patient–provider decision making when treating this complex disease.

## CONCLUSION

A gradual but sustained improvement in QOL along with diminishing of symptoms can be anticipated with curative‐intent multimodality salvage for RRC, while palliative treatments can be expected to ameliorate symptoms effectively and rapidly in the short term. Anatomical distribution of the recurrent disease, surgical resection margin, and patient gender all significantly influenced survivorship experience. This knowledge about longitudinal cancer survivorship has highlighted the additional sustained value of pursuing curative‐intent multimodality salvage for RRC when feasible beyond traditional survival outcomes.

## AUTHOR CONTRIBUTIONS

**Tarik Sammour:** Conceptualization; writing – original draft; investigation; methodology; writing – review and editing; formal analysis. **Oliver Peacock:** Conceptualization; investigation; methodology; writing – review and editing; formal analysis; data curation. **Brian K. Bednarski:** Writing – review and editing; formal analysis; methodology; investigation; data curation. **Arvind Dasari:** Investigation; methodology; writing – review and editing; formal analysis; data curation. **Prajnan Das:** Investigation; methodology; writing – review and editing; formal analysis; data curation. **Benny Johnson:** Methodology; writing – review and editing; formal analysis; data curation; investigation. **Grace L. Smith:** Formal analysis; data curation; writing – review and editing; methodology; investigation. **George J. Chang:** Conceptualization; investigation; methodology; writing – review and editing; supervision; formal analysis; data curation. **John Skibber:** Conceptualization; investigation; writing – review and editing; methodology; formal analysis; supervision; data curation. **Y. Nancy You:** Resources; supervision; data curation; project administration; formal analysis; writing – review and editing; writing – original draft; funding acquisition; investigation; conceptualization; methodology.

## FUNDING INFORMATION

This study is supported in part by the University of Texas MD Anderson Cancer Center—Duncan Family Foundation Cancer Survivorship Research Seed Money Grant (to YNY) and National Institutes of Health/National Cancer Institute grant CA016672 (University of Texas MD Anderson Cancer Center Support Grant). The grant funding agencies had no role in the design and conduct of the study; collection, management, analysis and interpretation of the data; preparation, review or approval of the manuscript; and decision to submit the manuscript for publication.

## CONFLICT OF INTEREST STATEMENT

We declare that we have no relevant conflicts of interest.

## ETHICS APPROVAL

The study was approved by the University of Texas MD Anderson Institutional Review Board.

## Data Availability

Research data are not shared.
